# Prenatal ultrasound, magnetic resonance imaging and therapeutic options for fetal thoracic anomalies: a pictorial essay

**DOI:** 10.1007/s00247-023-05681-y

**Published:** 2023-05-11

**Authors:** Pablo Caro-Domínguez, Teresa Victoria, Pierluigi Ciet, Estrella de la Torre, Ángel Chimenea Toscano, Lutgardo García Diaz, José Antonio Sainz-Bueno

**Affiliations:** 1 Pediatric Radiology Unit, Radiology Department, Hospital Universitario Virgen del Rocío, Avenida Manuel Siurot s/n, 41013 Seville, Spain; 2 Department of Pediatric Radiology, Massachusetts General Hospital, Harvard Medical School, Boston, MA USA; 3 Radiology and Nuclear Medicine Department, Erasmus, MC Rotterdam, The Netherlands; 4 Department of Pediatric Surgery, Hospital Universitario Virgen del Rocío, Seville, Spain; 5 Departmento de Medicina Materno-Fetal, Genética y Reproducción y Departamento deCirugía, Instituto de Biomedicina de Sevilla (IBIS), Hospital Universitario Virgen del Rocío, Universidad de Sevilla, Seville, Spain; 6 Department of Obstetrics and Gynecology, Valme University Hospital and Faculty of Medicine, University of Seville, Seville, Spain

**Keywords:** Anomaly, Fetus, Lung, Magnetic resonance imaging, Thorax, Treatment, Ultrasound

## Abstract

Congenital thoracic anomalies are uncommon malformations that require a precise diagnosis to guide parental counseling and possible prenatal treatment. Prenatal ultrasound (US) is the gold standard imaging modality to first detect and characterize these abnormalities and the best modality for follow-up. Fetal magnetic resonance imaging (MRI) is a complementary tool that provides multiplanar assessment and tissue characterization and can help estimate prognosis. Prenatal treatment is increasingly being used in fetuses with signs of distress and to potentially decrease morbidity and mortality. In this essay, the authors illustrate side-by-side US, MRI and therapeutic options for congenital thoracic anomalies in cases that presented to a tertiary pediatric hospital during the 7-year period 2014–2021. Entities included are congenital diaphragmatic hernia, congenital pulmonary airway malformation, bronchopulmonary sequestration, hybrid lesions, foregut duplications cysts and congenital lobar overinflation. Treatment options include maternal steroids, thoraco-amniotic shunt and fetal endotracheal occlusion. Recognition of typical findings in congenital thoracic anomalies is helpful to establish diagnosis, predict prognosis and plan perinatal treatment.

**Graphical Abstract:**

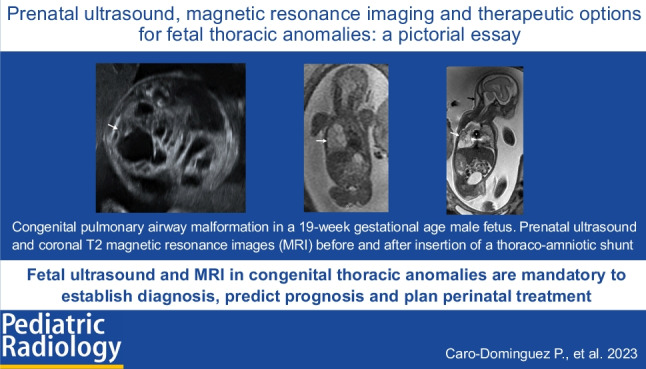

## Introduction

Congenital thoracic anomalies are uncommon entities that are usually diagnosed on routine prenatal ultrasound (US). They may present without complications but requiring regular follow-up during pregnancy, or might present with signs of distress requiring prompt perinatal treatment. Radiologists, gynecologists, neonatologists and surgeons should be familiar with the diverse imaging findings and treatment options for congenital thoracic anomalies to provide prompt diagnosis and appropriate treatment. This pictorial essay illustrates side-by-side imaging features and therapeutic options for the more common congenital thoracic anomalies based on cases that presented to a tertiary pediatric hospital during the 7-year period 2014 to 2021.

## Imaging approach

Diagnosis and treatment of congenital thoracic malformations have improved significantly in the last decades with routine fetal US screening and the introduction of fetal magnetic resonance imaging (MRI). Prenatal imaging provides valuable information about congenital malformations and prognosis, helping to improve perinatal care and guiding fetal therapies [1, 2].

Prenatal US is the imaging gold standard to evaluate fetal thoracic abnormalities due to its wide availability and non-invasive nature. Fetal US is performed throughout pregnancy to monitor the uterus, placenta and fetus. It offers real-time high-resolution images of the chest and obtains biometric measurements to compare with normative data. Doppler US allows determination of lesional vascular flow and possible signs of fetal distress [3]. In the last two decades, fetal MRI has become a valuable complementary tool in the imaging armamentarium of fetal thoracic anomalies, particularly in congenital diaphragmatic hernia [4–8]. The advantages of MRI in evaluating the fetal chest are better anatomical definition due to its multiplanar capability, characterization of different tissues and the precise quantification of pulmonary volumes [9]. Visualization of the fetal chest with MRI is not limited by maternal obesity or fetal lie as is sometimes the case with US. MRI also provides a large field of view of the fetal thorax and its surroundings, which may allow prompt identification of the signs of fetal distress. Despite these advantages, the use of fetal MRI is not standard of care for the assessment of congenital lung abnormalities. Limiting factors include lack of specific training in some countries, cost and scan time [10].

Assessment of fetal thoracic anomalies with MRI can be performed with 1.5- or 3-tesla MRI machines using standard single-shot fast spin echo T2 sequences of the uterus and fetal body in the three orthogonal planes (7-mm slice thickness for the uterus, 3-mm thickness for the body) and at least one T1 turbo field echo sequence of the fetal body (mainly coronal 3-mm thickness). Diffusion-weighted imaging can be used to assess the cellularity of lesions. Restricted diffusion (high signal on high *b* values and low signal on apparent diffusion coefficient maps) is identified in highly cellular solid tumors (i.e. malignancies) [2]. Susceptibility-weighted echo planar MRI can be used to identify calcium or hemorrhage within the lesions (low signal) [2]. A summary of our MRI protocol is given in Table 1.

**Table 1:** Magnetic resonance imaging protocol to study congenital lung lesions (1.5 tesla system; Philips Achieva, Eindhoven, The Netherlands)

	Single-shot T2 TSE of the maternal uterus	Single-shot T2 TSE of the fetal chest	T1 FFE gradient	Diffusion-weighted Imaging EPI	T2 FFE gradient
Information obtained	Position of the fetus, volume of amniotic fluid, position and signal intensity of the placenta, cervix	Situs, position of the heart and aortic arch. Lung signal intensity and volume, diaphragm integrity, signal intensity of lesions, size, cystic component, mass effect	Integrity of the diaphragm, position of the liver and meconium	Cellularity of lesions (*b* values 0, 1,000)	Hemosiderin, calcium within a lesion
Orientation	Coronal Sagittal Axial	Axial Coronal (Sagittal)	Coronal (Axial)	Axial	Axial
Repetition time (ms)	Shortest	Shortest	10	Shortest	Shortest
Echo time (ms)	95	105	4.6	Shortest	23
Field of view (mm)	382 × 396 × 191	435 × 481 × 93	375 × 304 × 119	400 × 375 × 195	374 × 299 × 79
Voxel size (mm)	1.16 × 1.19	1.16 × 1.19	1.5 × 2	3 × 3	0.9 × 1.12
Slice thickness (mm)	7	3	5	5	4
Slice gap (mm)	1	0	1	0.6	0
NSA	1	1	2	3	1
Flip angle (degrees)	90	90	15	90	18
Scan time (s)	70 per plane	80 per plane	60 per plane	90 per plane	150

*EPI* echo planar imaging, *FFE* fast field echo, *NSA* number of signal averages/acquisitions, *TSE* turbo spin echo

MRI has shown high diagnostic accuracy (98%) in the prenatal diagnosis of congenital thoracic malformations [8]. In a study of 74 fetuses with thoracic abnormalities by Levine et al. [5], MRI provided additional information to US in 28 patients (37.8%) and changed management and expected prognosis in 6 out of 74 fetuses (8.1%). Some groups have also advocated the use of late third trimester prenatal MRI in preparation for possible early postnatal intervention, thus obviating the use of radiation by means of postnatal CT [8].

In our practice, when a fetal thoracic lesion is detected, both US and MR are performed to evaluate its size, margins, vascularity, mass effect on surrounding organs, integrity of the diaphragm (if involved) and to exclude signs of hydrops.

## Fetal treatment

Most fetuses diagnosed with a fetal thoracic mass will not need treatment in utero, only requiring close sonographic follow-up through the remainder of the gestation. Invasive procedures, like amniocentesis, can be performed in selected cases to obtain genetic information (karyotype, arrays +/− exome).

Prenatal treatment includes maternal intramuscular injection of steroids for predominantly solid large congenital pulmonary airway malformations (pleuro-amniotic shunt [for congenital pulmonary airway malformation with large cysts and resultant mass effect or hydrops] and fetal endoluminal tracheal occlusion [for right or severe left congenital diaphragmatic hernia]). The ex utero intrapartum procedure may be considered a form of delivery for upper airway obstruction, large lung lesions or during prenatal surgery [11–20]. Fetal endoluminal tracheal occlusion is a minimally invasive procedure consisting of temporary occlusion of the trachea with a balloon, using an endoscopic approach that is removed or deflated at 34 weeks of gestation [16]. The balloon seals the airway, preventing fluid from exiting the lungs, causing an increase in transpulmonic pressure and accelerating pulmonary growth [16].

## Types of congenital thoracic anomalies seen at our institution

Our institutional research ethics board approved this review. We retrieved 196 fetal MRI studies from our picture archiving and communication system during the time period from February 2014 to December 2021. Of these, 44 were obtained to evaluate a thoracic congenital anomaly. Final diagnoses were as follows: 16 congenital diaphragmatic hernia (12 left, 4 right), 13 congenital pulmonary airway malformations, 4 bronchopulmonary sequestrations including one associated with congenital diaphragmatic hernia, 7 hybrid lesions (features of bronchopulmonary sequestration and congenital pulmonary airway malformation) including 2 associated with congenital diaphragmatic hernia, 2 bronchogenic cysts including 1 associated with congenital pulmonary airway malformation, 1 esophageal duplication cyst and 1 congenital lobar overinflation. Clinical findings are summarized in Table 2.

**Table 2:** Summary of clinical findings of 44 fetuses with sonographic suspicion of congenital thoracic anomalies who required fetal magnetic resonance imaging

	*n*
Final diagnosis based on postnatal imaging and pathology	
CDH	16 (12 left, 4 right)
CPAM	13
BPS	4 (1 with CDH)
Hybrid lesion	7 (2 with CDH)
Bronchogenic cyst	2 (1 with CPAM)
Esophageal duplication cyst	1
Congenital lobar overinflation	1
Treatment	
No treatment	28
Maternal steroids	8
Thoraco-amniotic shunt	1
Fetal endotracheal occlusion	2
Termination of pregnancy	3 (severe CDH)
Survival at 1st year of life	37
Exitus	4 (3 CDH, 1 CPAM)

*BPS* bronchopulmonary sequestration, *CDH* congenital diaphragmatic hernia, *CPAM* congenital pulmonary airway malformation

## Congenital diaphragmatic hernia

Congenital diaphragmatic hernia is a relatively uncommon congenital malformation characterized by a defect in the diaphragm, leading to the herniation of abdominal contents into the thoracic cavity. They are predominantly left-sided (85%), but can also be right-sided (13%), bilateral (2%) or intrapleural and mediastinal [21]. The most common intrapleural congenital diaphragmatic hernia is the Bochdalek hernia, resulting in mass effect manifested as contralateral cardiomediastinal shift and pulmonary hypoplasia. When retrosternal, it is called a Morgagni hernia [21].

**Fig. 1:**
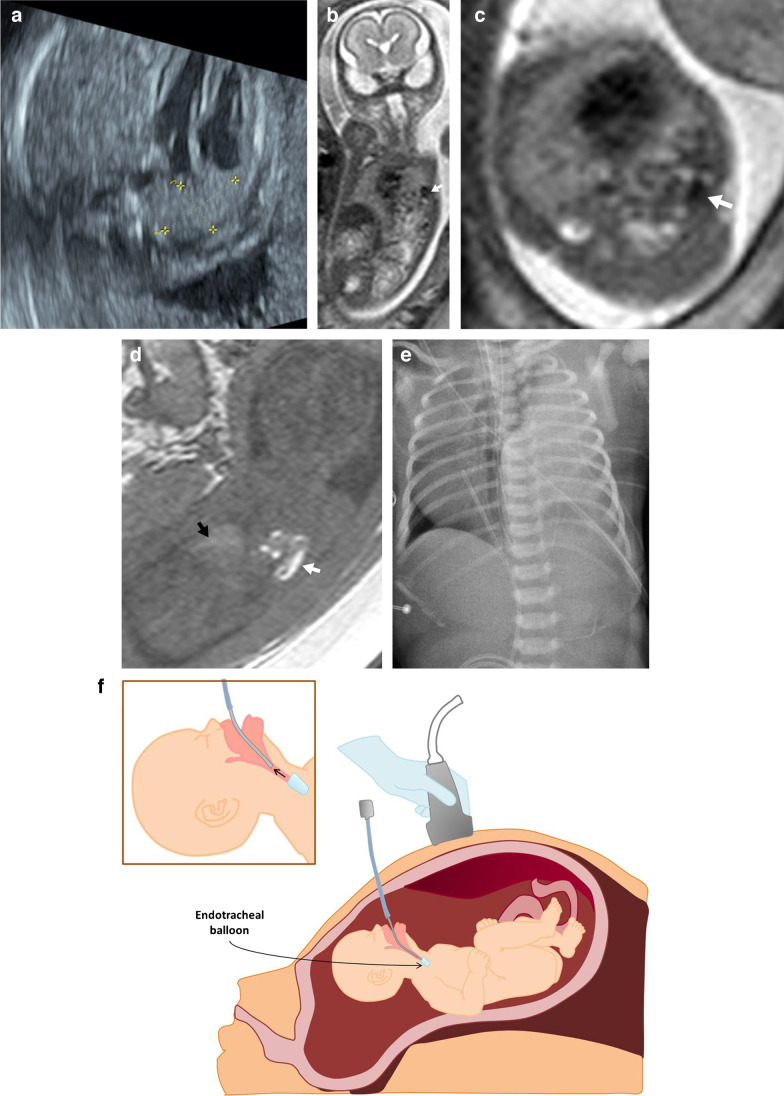
A left Bochdaleck diaphragmatic hernia in a 20-week gestational age female fetus. **a** An axial ultrasound image used to predict prognosis based on the lung-to-head ratio shows the volume of the left lung being measured (*cursors*). **b**–**d** Amniocentesis and fetal magnetic resonance imaging were performed in the same week. Single-shot T2 coronal (**b**) and axial (**c**) images show multiple bowel loops in the left hemithorax due to congenital diaphragmatic hernia. Coronal T1 image (**d**) demonstrates meconium with high T1 signal within the herniated bowel loops in the left hemithorax (*white arrow*). Note the liver (*black arrow*) with high T1 signal below the intact right hemidiaphragm. **e** The first radiograph obtained after birth shows increased density in the left hemithorax due to the herniated bowel loops that are still non-aerated. Surgery performed on the first day of life confirmed a left Bochdaleck diaphragmatic hernia. **f** A drawing illustrates the fetal endoscopic tracheal occlusion procedure

Congenital diaphragmatic hernia is usually identified at routine US during the second trimester of gestation [22]. Fetal sonographic findings of congenital diaphragmatic hernia include intrathoracic liver (associated with a worse prognosis) with visualization of the hepatic veins coursing from the abdomen into the thorax and the presence of loops of bowel in the thorax. Sometimes, there are additional organs herniated into the fetal chest, including the spleen and kidney. Determination of cardiomediastinal shift and degree of lung hypoplasia is important. Ultrasound can be used to predict prognosis using the observed-to-expected lung-to-head ratio (Fig. 1). A low observed-to-expected lung-to-head ratio (<25%) is a predictor of poor postnatal outcome [23].

MRI allows better visualization of the diaphragmatic defect and herniated contents compared to US [2]. T1 sequences are particularly helpful in evaluating congenital diaphragmatic hernia because they confirm the presence of intrathoracic liver and meconium filled bowel, given the high T1 signal of liver and meconium (Fig. 1). More significantly, MRI is useful in evaluating lung volumes and degree of lung hypoplasia [6]. Total fetal lung volume is obtained as the sum of the fetal lung areas on each slice multiplied by the slice thickness [24]. This lung volume is then compared to the expected lung volume for gestational age. The observed-to-expected fetal lung volume ratio reflects the severity of lung hypoplasia; fetuses with observed-to-expected total fetal lung volume of less than 25% have a higher likelihood of requiring extracorporeal membrane oxygenation or developing severe pulmonary hypertension after birth, with higher mortality rates [9]. The herniated liver volume is another prognostic factor, calculated by dividing the percentage of herniated liver by total fetal lung volume, with higher numbers being associated with worse postnatal prognosis [25].

Mild and moderate left congenital diaphragmatic hernia can be followed with US throughout pregnancy and surgically corrected after birth. For cases of right and severe left-sided congenital diaphragmatic hernia, prenatal treatment with fetal endoscopic tracheal occlusion has shown to improve outcome.

## Congenital pulmonary airway malformation

Congenital pulmonary airway malformation is defined as an abnormal development of the bronchoalveolar unit due to hamartomatous proliferation. They commonly affect a single lobe, communicate with the bronchial tree and have pulmonary blood supply and drainage [3]. Histopathological postnatal analysis can differentiate them into five types. At prenatal imaging, congenital pulmonary airway malformation can be divided into macrocystic or microcystic/solid type depending on whether the cysts are larger or smaller than 5 mm [3].

**Fig. 2:**
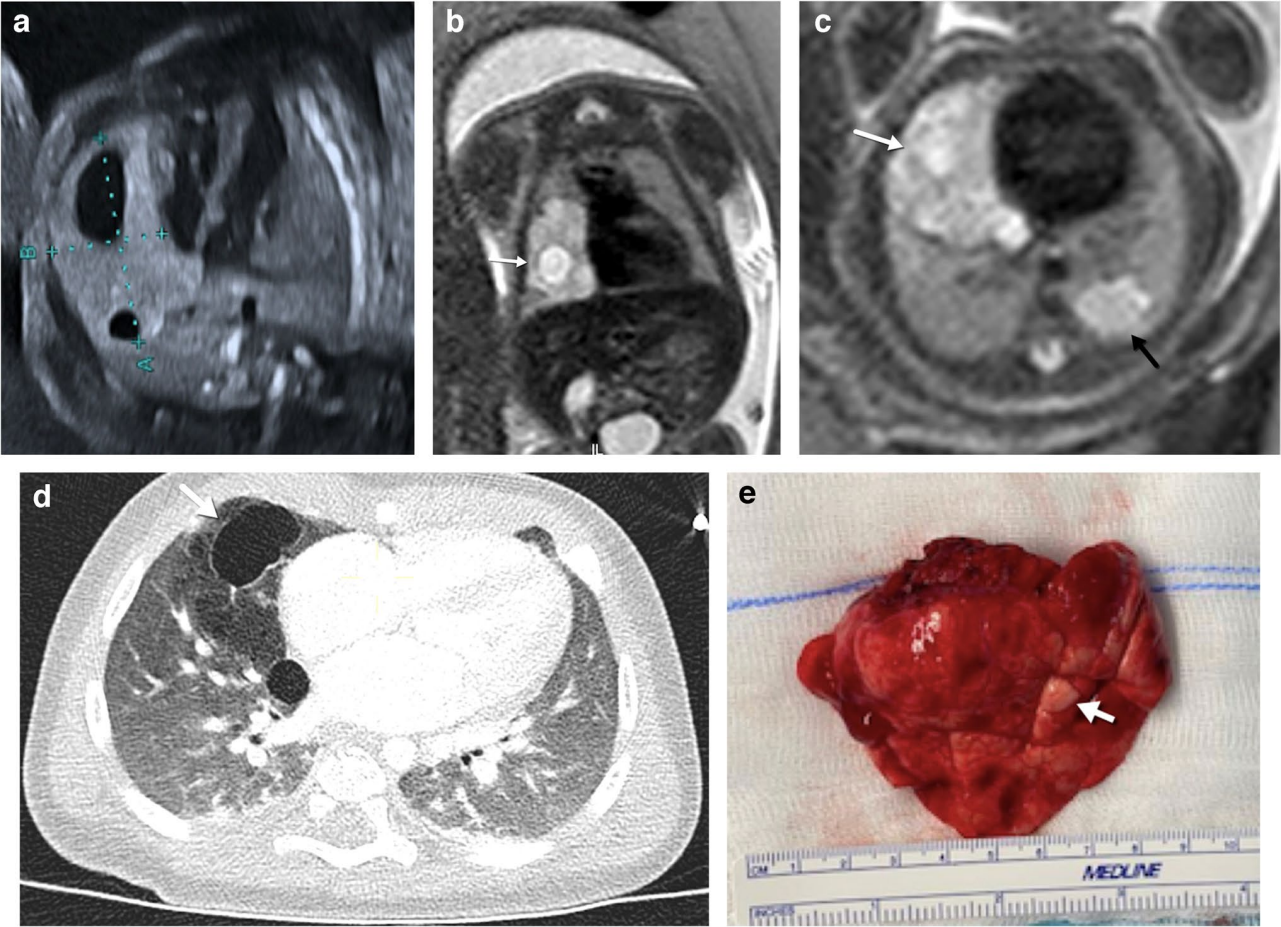
A congenital pulmonary airway malformation in a 22-week gestational age male fetus. **a** Axial ultrasound image of the chest shows a hyperechoic lung lesion with two macrocystic lesions in the middle lobe of the right lung. **b**, **c** Fetal magnetic resonance imaging was performed the same week. Single-shot T2 coronal (**b**) and axial (**c**) images confirm the presence of a lung mass with multiple cysts in the middle lobe and a second lesion in the left lower lobe. The lesion located in the middle lobe has a dominant macrocyst (*white arrows*), whereas the lesion in the left lower lobe contains microcysts (*black arrow in ***c**). No signs of hydrops or mediastinal shift are noted. These lesions were treated with intramuscular injections of steroids to the mother. **d** An axial postnatal computed tomography image (lung windows) obtained at 6 months of age confirms the presence of a cystic lung lesion in the middle lobe. **f** Surgical photograph after middle lobectomy demonstrates congenital pulmonary airway malformation type 1 with multiple cysts (*arrow*)

**Fig. 3:**
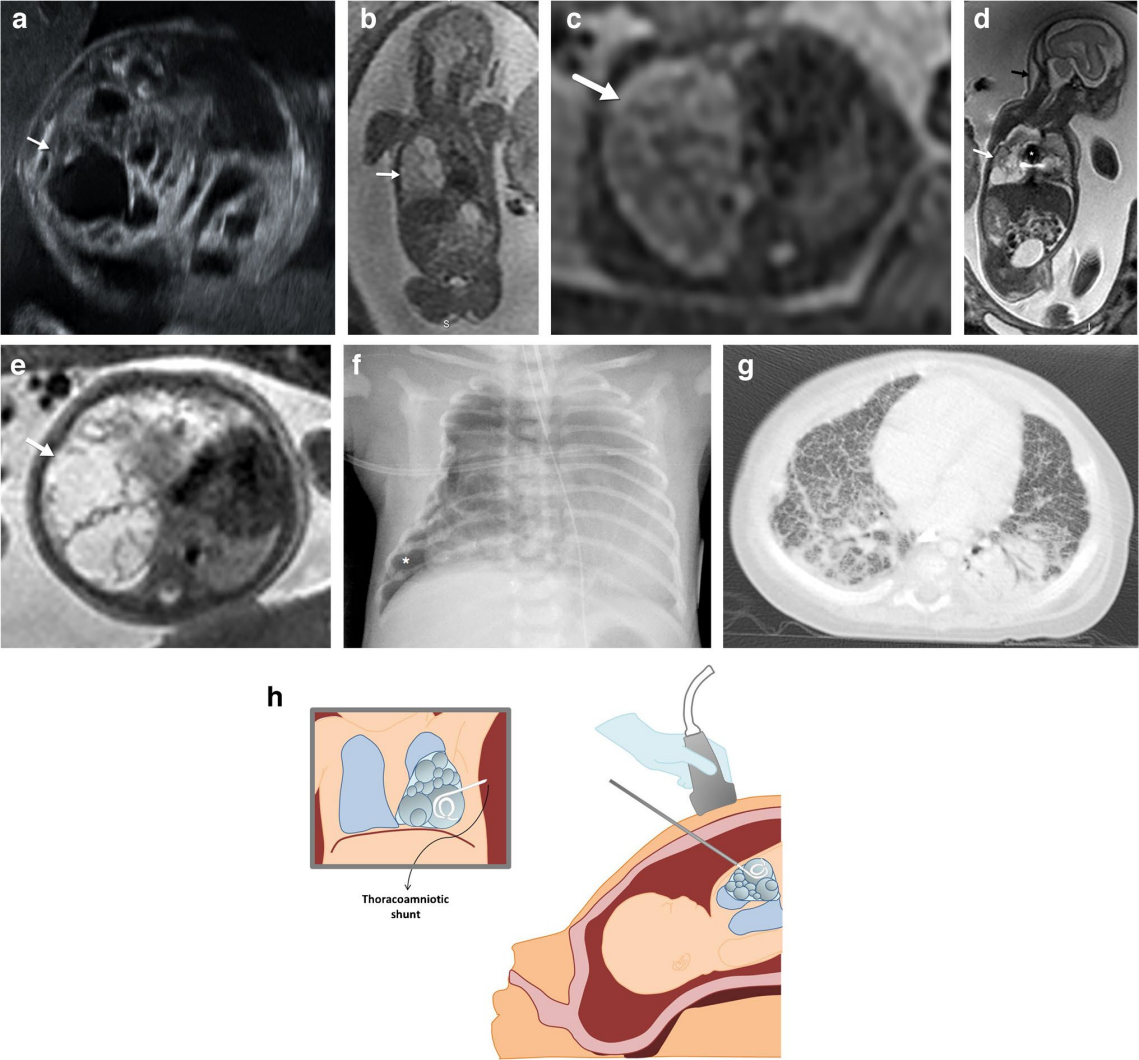
A congenital pulmonary airway malformation in a 19-week gestational age male fetus. **a** Axial ultrasound image shows a multiloculated cystic lesion in the right middle lobe displacing the mediastinum to the left. **b**,** c** Fetal magnetic resonance imaging (MRI) was performed the same week. Single-shot T2 coronal (**b**) and axial (**c**) images show a large heterogeneous lung mass (*arrows*) occupying the right hemithorax with microcysts displacing the mediastinum. Follow-up MRI was performed at 25 weeks of gestation after developing hydrops which was treated with steroids and the subsequent placement of a thoraco-amniotic shunt (*asterisk*). **d, e** Coronal (**d**) and axial (**e**) T2 single-shot MRI show a large mass in the right hemithorax with mediastinal shift and subtle inferior displacement of the ipsilateral diaphragm. Mild skin thickening and ascites are noted in keeping with early hydrops. **f** Anteroposterior chest radiograph performed on the first day of life shows a right pneumothorax (*asterisk*) and a right chest wall deformity secondary to the shunt placement. Surgery was performed in the second week of life due to respiratory distress and demonstrated congenital pulmonary airway malformation. **g** An axial computed tomography (CT) image (lung windows) obtained at the age of 6 months shows bilateral reticular interstitial pattern. This infant died 1 month after the CT due to respiratory failure. **h** A drawing illustrates the thoraco-amniotic shunt procedure

Congenital pulmonary airway malformation is usually first diagnosed in the second trimester by means of US, as a predominantly homogeneous lesion with solid and/or cystic components that may displace the surrounding structures (Fig. 2). On US, macrocystic lesions show larger cysts surrounded by echogenic tissue (Fig. 3), while microcystic lesions are predominantly hyperechogenic solid lesions which may contain visible microcysts [3]. These lesions may become isoechoic to the surrounding lung parenchyma during the third trimester [3]. On MRI, macrocystic lesions appear as unilocular or multilocular high T2 signal cystic lesions of variable size with thin walls. Some lesions containing predominantly T2 hypointense components correlate with congenital pulmonary airway malformation of primitive histology and are more resistant to steroid therapy when compared to the traditional congenital pulmonary airway malformations [26].

Ultrasound is the preferred imaging technique for follow-up of these lesions and their potential complications. The best prognostic indicator for a congenital pulmonary airway malformation is the congenital pulmonary airway malformation volume ratio, which is a calculated volume based on US measurements obtained in three dimensions of the mass at presentation. The volume is then divided by the head circumference (thereby correcting for gestational age) [27]. The prognosis of congenital pulmonary airway malformation is usually favorables with spontaneous decrease in size in many cases and apparent disappearance of some lesions [28–31]. Some theories to explain this involution include decompression into the bronchial tree and decreased blood supply. Although some masses may appear to resolve completely prenatally, CT after delivery often identifies a residual lesion.

Small congenital pulmonary airway malformations with a volume ratio of less than 1.4 can be followed through gestation using US without the need to treat. In solid lesions with a volume ratio of more than 1.4, maternal injection of steroids is recommended, to decrease the size of the lesions and to encourage fetal lung maturation [19]. Congenital pulmonary airway malformation lesions with relatively large cysts causing fetal distress can be treated with US-guided thoraco-amniotic shunting (Fig. 3) or thoracocentesis [15]. If the congenital pulmonary airway malformation is predominantly solid and signs of in utero fetal distress develop, surgical resection might be considered [29]. Fetal distress is manifested as the development of hydrops.

## Bronchopulmonary sequestration

Bronchopulmonary sequestration is defined as a malformation of nonfunctioning bronchopulmonary tissue separated from the bronchial tree and supplied by the systemic circulation, usually from the aorta or one of its major branches [3]. There are two types, intralobar which is not covered by separate visceral pleura and drains into the pulmonary veins and extralobar, which has its own visceral pleural cover and drains into systemic veins [3].

**Fig. 4.**
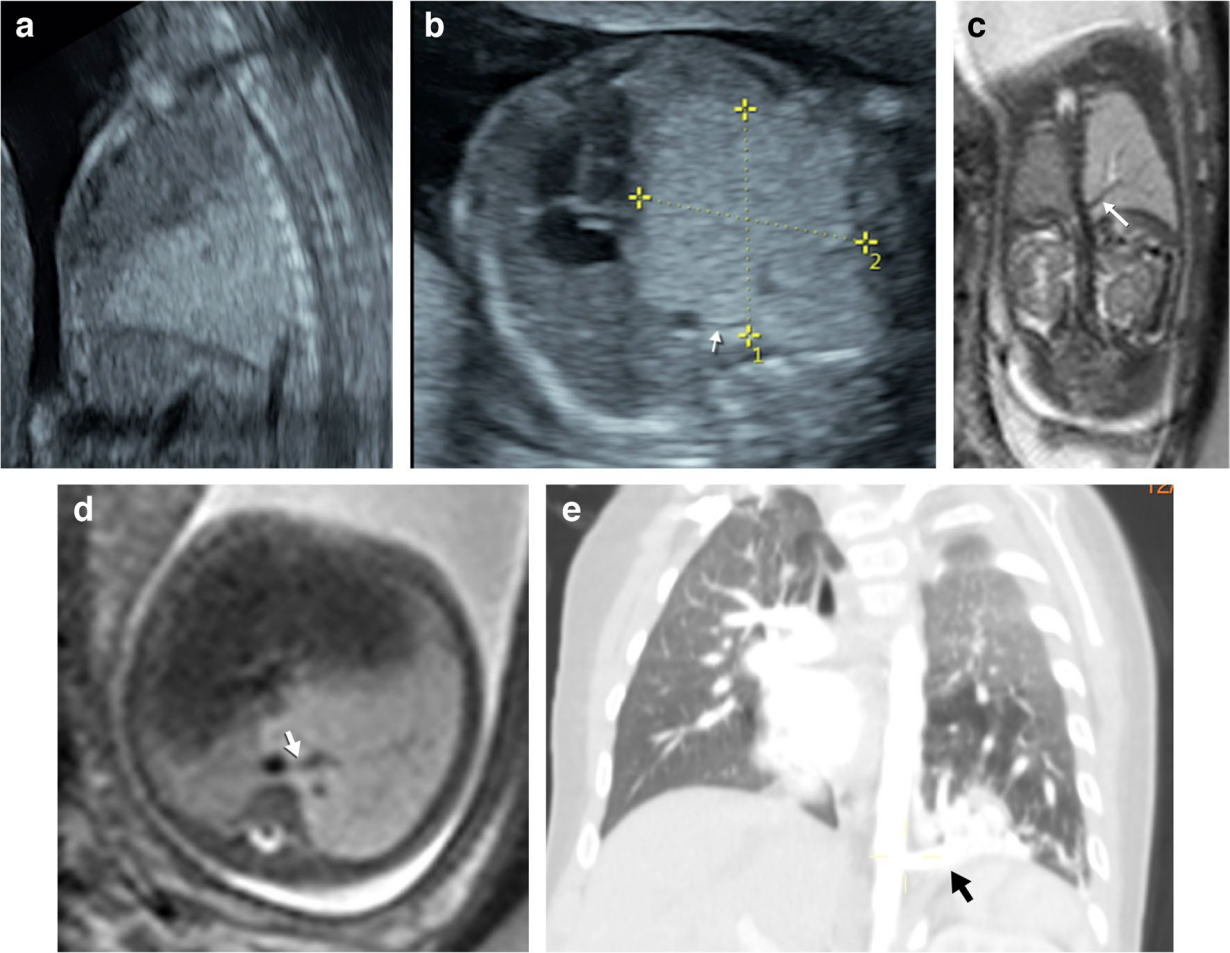
An intralobar bronchopulmonary sequestration in a 20-week gestational age female fetus. **a**, **b** Sagittal (**a**) and axial (**b**) ultrasound images demonstrate a hyperechoic lung lesion in the left lower lobe (*calipers* in **b**) mildly displacing the heart to the right with a linear hypoechoic structure entering it, suggestive of a feeding vessel (*arrow* in **b**). **c**, **d** Fetal magnetic resonance imaging was performed at 21 weeks of gestation. Single-shot T2 coronal (**c**) and axial (**d**) images confirm the presence of a large lesion in the left lung, with homogeneous high T2 signal and a feeding vessel arising from the descending thoracic aorta (*arrow* in **d**). **e** Contrast-enhanced coronal computed tomography image on lung window obtained at 6 months of age confirms the lesion in the left lung base and the feeding vessel  (*arrow*). Surgery demonstrated intralobar sequestration

Bronchopulmonary sequestration is usually detected on the second trimester prenatal US as a supradiaphragmatic echogenic triangular solid mass located between the lower lobe (usually the left) and the diaphragm with a feeding vessel arising from the descending thoracic aorta entering the mass (Fig. 4). Less frequently (10%), it can be identified below the diaphragm and mimic an adrenal lesion (hemorrhage or neuroblastoma) [3].

**Fig. 5:**
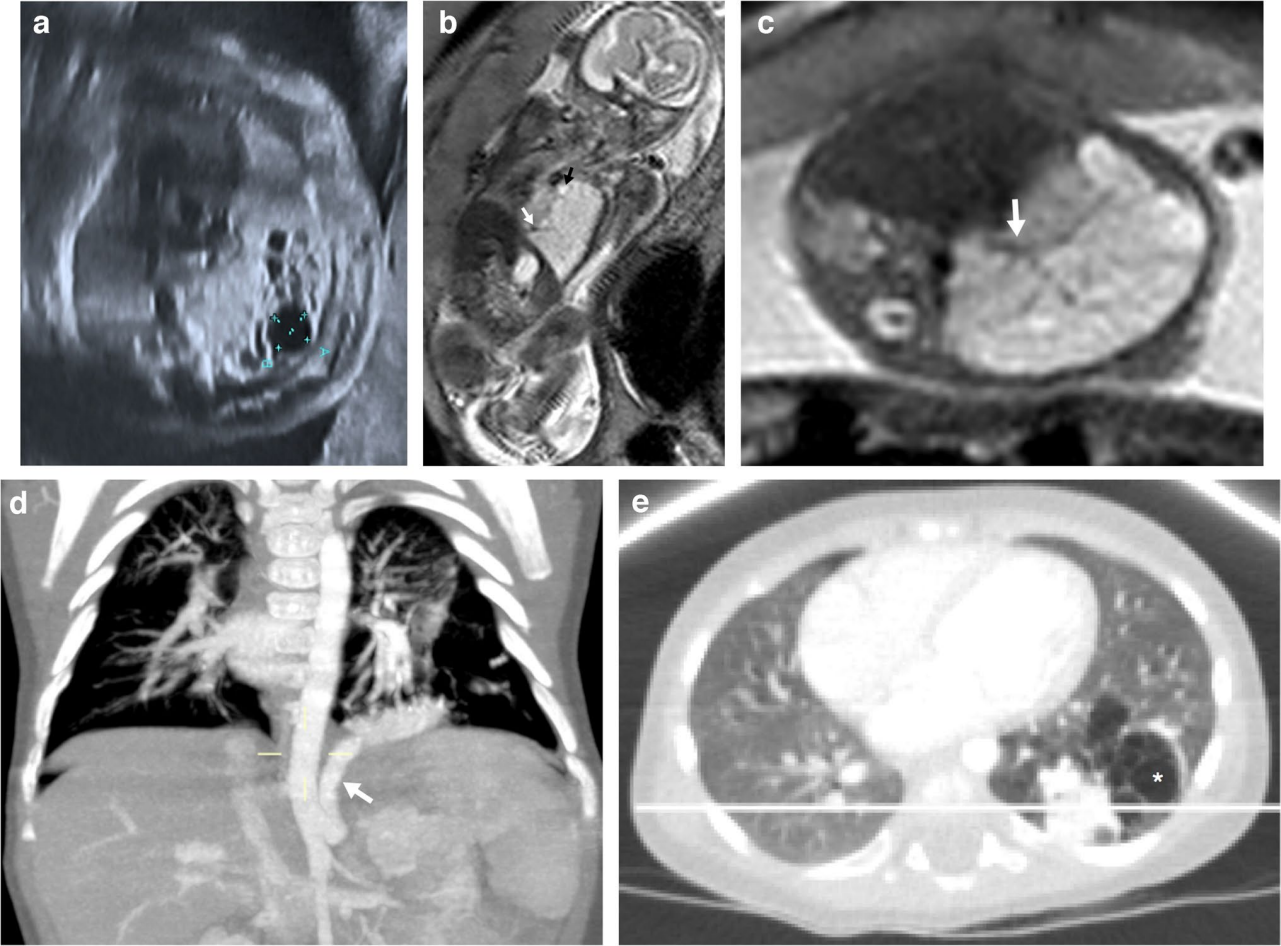
A hybrid lesion (intralobar sequestration and congenital pulmonary airway malformation) in a 21-week gestational age female fetus. **a** An axial ultrasound image demonstrates a hyperechoic lesion in the left lung base with multiple cysts within it (*calipers*). **b**, **c** Fetal magnetic resonance imaging was performed at 22-weeks gestational age. Single-shot T2 coronal (**b**) and axial (**c**) images show a large homogeneous lesion in the left lung, with high signal intensity on T2. There is a feeding vessel (*white arrows*) arising from the descending thoracic aorta and small cysts in the periphery of the lung (*black arrow in ***b**), suggestive of a hybrid lesion. **d**, **e** Coronal maximum intensity projection (**d**) and axial lung window (**e**) computed tomography images obtained at 5 months of age confirm the lesion in the left lung base and feeding vessel (*arrow* in **d**) and multiple cysts (*asterisk in ***e**). Surgery demonstrated a hybrid lesion (intralobar sequestration and congenital pulmonary airway malformation)

Bronchopulmonary sequestration appears on fetal MRI as a solid lesion with well-defined margins homogeneously hyperintense on T2, with a linear T2 hypointense feeding vessel arising from the aorta (or one of the abdominal arteries) entering into the mass. A hybrid lesion (with features of congenital pulmonary airway malformation and sequestration) is suspected if the lesion has cystic components and a feeding vessel [8] (Fig. 5).

Bronchopulmonary sequestration can partially or completely regress (50% disappear in utero), with progressive decrease of T2 signal intensity and size [3]. Large lesions can cause mediastinal shift and compress the thoracic veins, leading to hydrops. In severe cases where fetal respiratory exchange may be compromised, an ex utero intrapartum treatment procedure may be considered [20]. Pleural effusion may occur in 6–10% of cases; these usually have a small vascular pedicle that may have torted, leading to lymphatic spillage and resulting in pleural effusion. If tension pleural effusion or signs of distress appear (i.e. hydrops), thoraco-amniotic shunts can be placed in the fetal cavity [15].

## Foregut duplication cysts

**Fig. 6:**
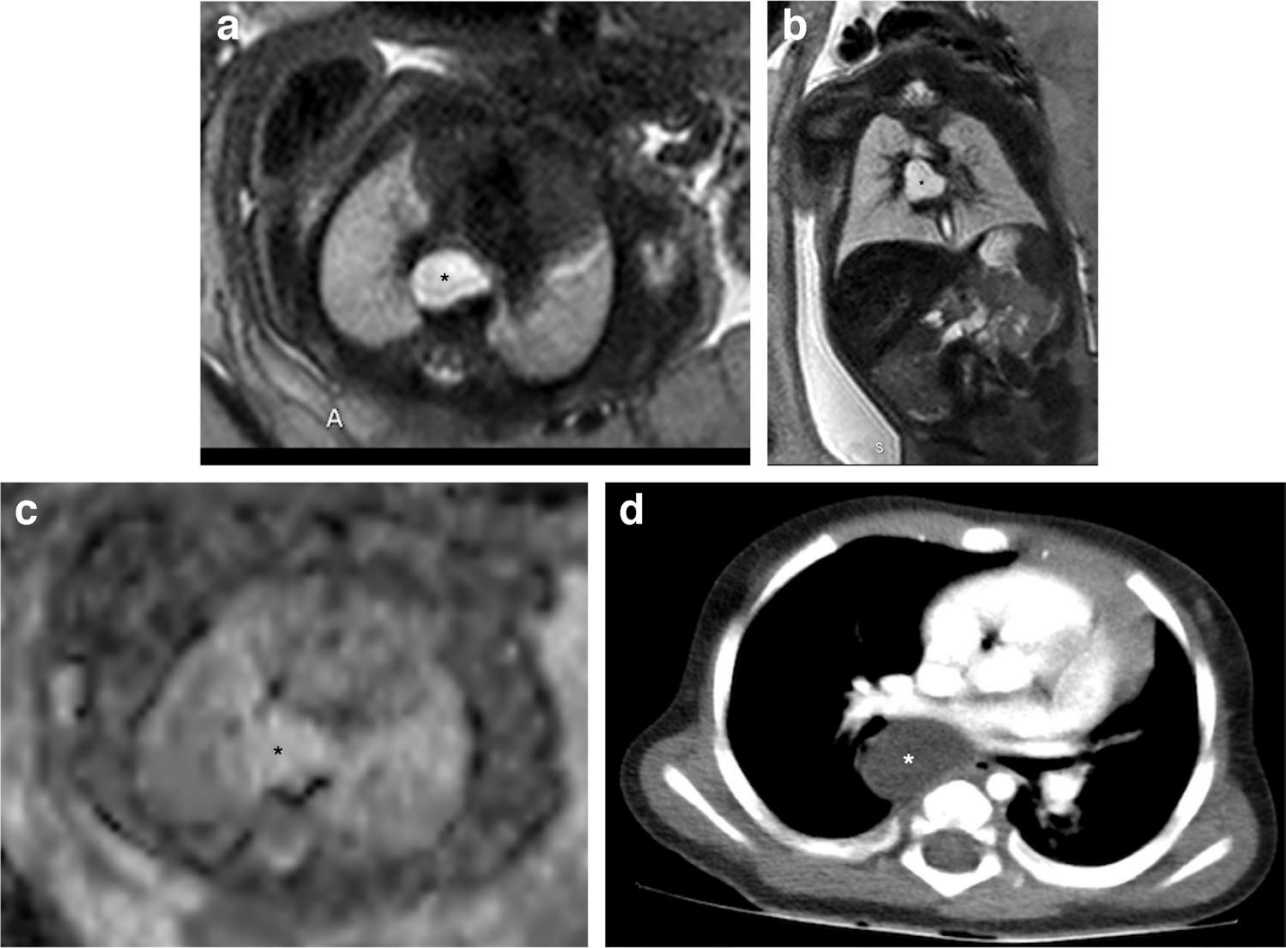
A bronchogenic cyst in a 27-week gestational age female fetus. **a**–**c** Single-shot T2 axial (**a**) and coronal (**b**) and axial apparent diffusion coefficient (ADC) (**c**) magnetic resonance images show a well-circumscribed homogeneous lesion (*asterisks*) in the right perihilar region, posterior to the right pulmonary veins and below the carina. It shows homogeneous high signal intensity on T2 and ADC map in keeping with a cystic lesion with clear contents, consistent with a bronchogenic cyst. **d** A post-contrast axial computed tomography image obtained at age 7 months confirms a homogeneously hypoattenuating cystic lesion in the mediastinum, posterior to the right pulmonary veins and left atrium and below the carina (*asterisk*). Surgery confirmed a bronchogenic cyst

**Fig. 7:**
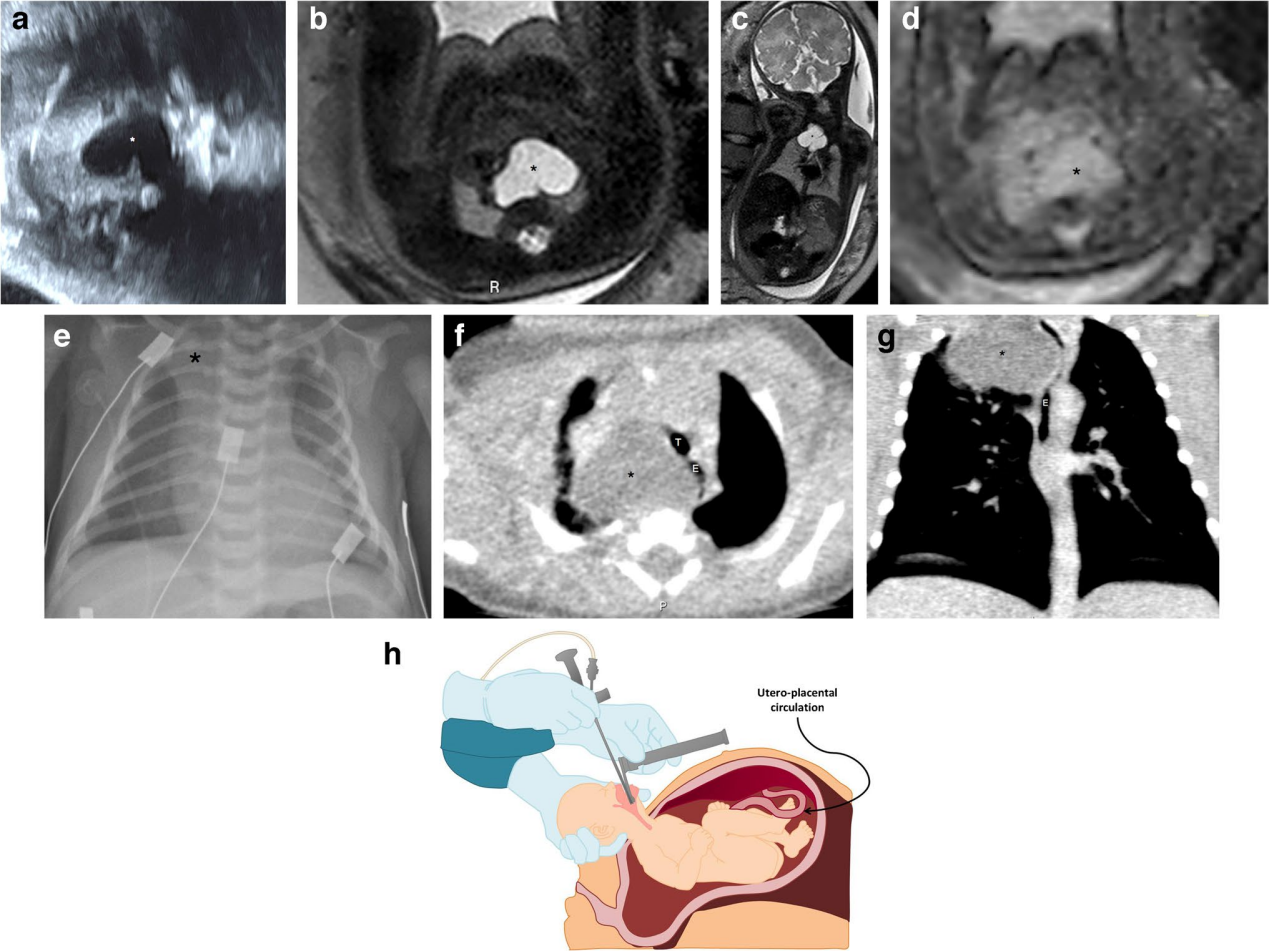
An esophageal duplication cyst in a female fetus. **a** An axial ultrasound image of the upper mediastinum at 35 weeks of gestation demonstrates an anechoic well-defined unilocular cystic lesion (*asterisk*). **b**–**d** Fetal magnetic resonance imaging was performed at 36 weeks of gestation. Single-shot T2 axial (**b**) and coronal (**c**) images show a well-circumscribed homogeneous lesion (*asterisks*) in the superior mediastinum, in close contact with the trachea and thymus. The lesion, which contains a few thin septations, shows homogeneously hyperintense signal intensity on T2 and apparent diffusion coefficient map (**d**) in keeping with a cystic lesion with clear contents. **e** A frontal chest radiograph performed on the first day of life demonstrates widening of the upper mediastinum by the lesion (*asterisk*). **f**, **g** Axial (**f**) and coronal (**g**) computed tomography images obtained at the age of 3 weeks confirm a homogeneously hypodense cystic lesion in the upper mediastinum, to the right of the trachea (*T*) and esophagus (*E*), without significant displacement. Surgery confirmed an esophageal duplication cyst. **h** A drawing illustrates ex utero intrapartum treatment

Foregut cysts are lined with ciliated columnar epithelium, and can cause airway obstruction due to compression of the trachea or bronchi [32]. They include bronchogenic cysts (the most common) and enteric and neurogenic cysts. Most often, the generic term foregut duplication cysts and their relationship to other structures are used due to the difficulty in differenting between them. Foregut duplication cysts appear on prenatal US as unilocular or multilocular thin-walled anechoic cysts [3]. When located at the pulmonary hilum or close to the tracheo-bronchial tree, a bronchogenic cyst is suspected (Fig. 6). Cysts close to the esophagus suggest esophageal duplication cysts (Fig. 7), while paravertebral cysts suggest neurogenic cysts, which might be associated with vertebral anomalies/communication with the spinal canal [3].

Fetal MRI shows a well-defined cystic lesion with high T2 signal and T2 shine-through on diffusion-weighted imaging [8]. Foregut duplication cysts have good prognosis, and only in rare cases do they cause compression of the adjacent structures. Conservative management during pregnancy is sufficient in the vast majority of cases. If compression of the upper airway is present, ex utero intrapartum treatment can be considered [20]. This procedure allows the axial establishment of a stable airway before clamping the umbilical cord, which maintains placental function until the airway is secure.

**Fig. 8:**
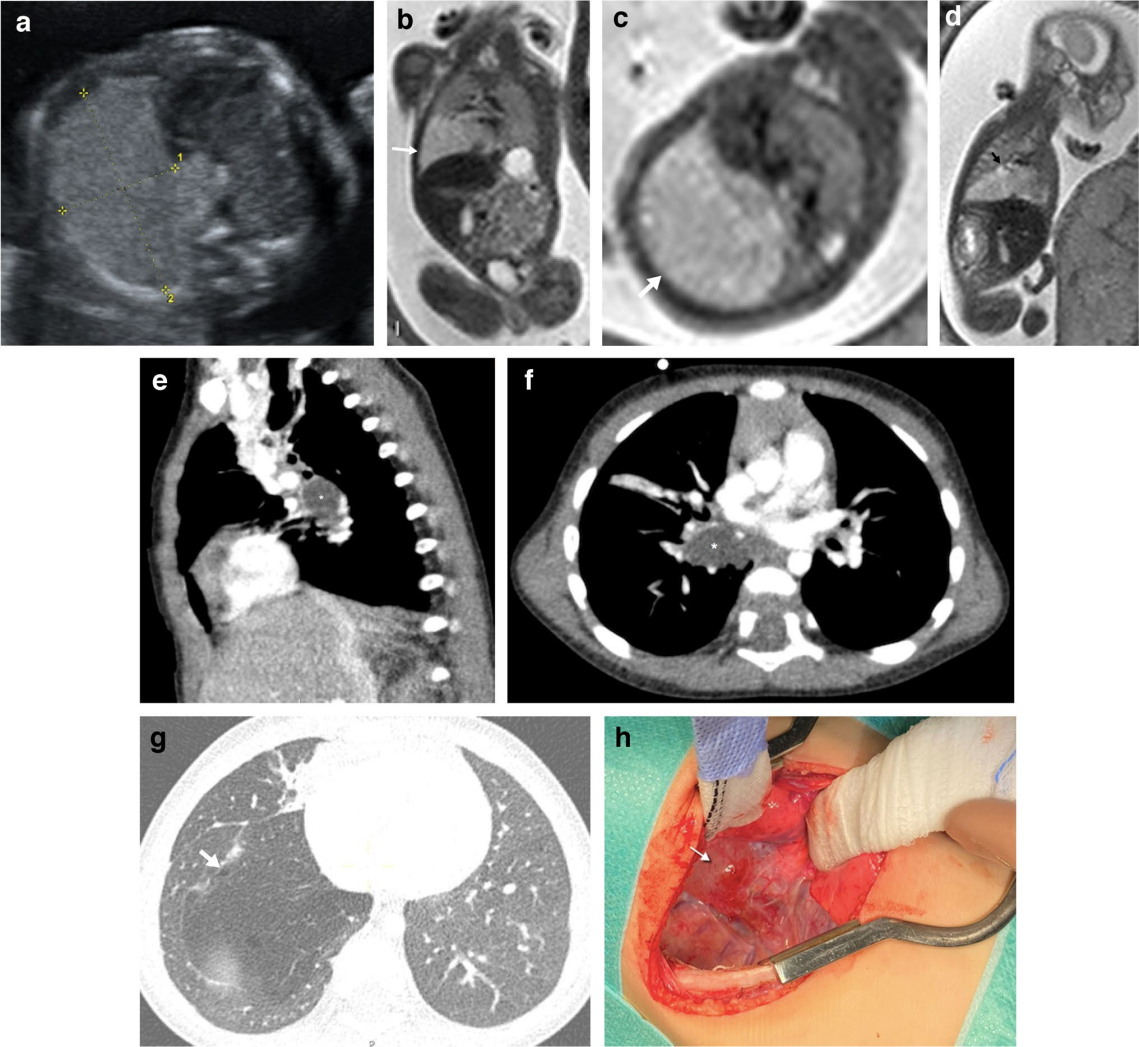
**a**–**d** Bronchogenic cyst and congenital pulmonary airway malformation in a 20-week gestational age male fetus. **a **An axial ultrasound image of the chest shows a large homogeneous hyperechoic lesion in the right lung base (*calipers*). **b**–**d** Single-shot T2 coronal (**b**), axial (**c**) and sagittal (**d**) magnetic resonance images show a large homogeneous lesion in the right lung base of high signal intensity on T2 (*white arrows in ***b** and **c**), with small cysts in the periphery (*black arrow in ***d**). No abnormal feeding vessel or hilar cystic lesion was identified. **e**–**g** Sagittal (**e**) and axial computed tomography images on mediastinal (**e**, **f**) and lung window (**g**) settings obtained at 6 months of age show a homogeneously hypodense cystic lesion in the right pulmonary hilum (*asterisks*) with atelectasis in the middle lobe and hyperinflation in the left lower lobe, with small peripheral cysts (*arrow in ***g**). **h** Photograph obtained at surgery demonstrates a bronchogenic cyst (*arrow*) associated with congenital pulmonary airway malformation

The association of foregut duplication cysts with other foregut malformation lesions such as bronchopulmonary sequestration and congenital pulmonary airway malformation should be considered and has been described in multiple case reports [33–35]. These cases support the current thinking that congenital pulmonary airway malformation/sequestration/bronchogenic cysts form a spectrum of abnormal development of the airways with obstructive dysplastic changes [36, 37]. In our cohort of patients, there was overlap/association of these pathologies (congenital diaphragmatic hernia, congenital pulmonary airway malformation, bronchopulmonary sequestration, bronchogenic cyst) in 20% of cases (9/44), presumably related to the common embryology and pathophysiology of these lesions. These pathologies can be concomitant, and if missed on prenatal imaging, they may become apparent on postnatal chest CT (Fig. 8).

## Congenital lobar overinflation

Congenital lobar overinflation, previously known as congenital lobar emphysema, is an alveolar hyperexpansion without wall destruction, distal to a discrete bronchial obstruction, that most commonly affects the left upper lobe [3].

**Fig. 9:**
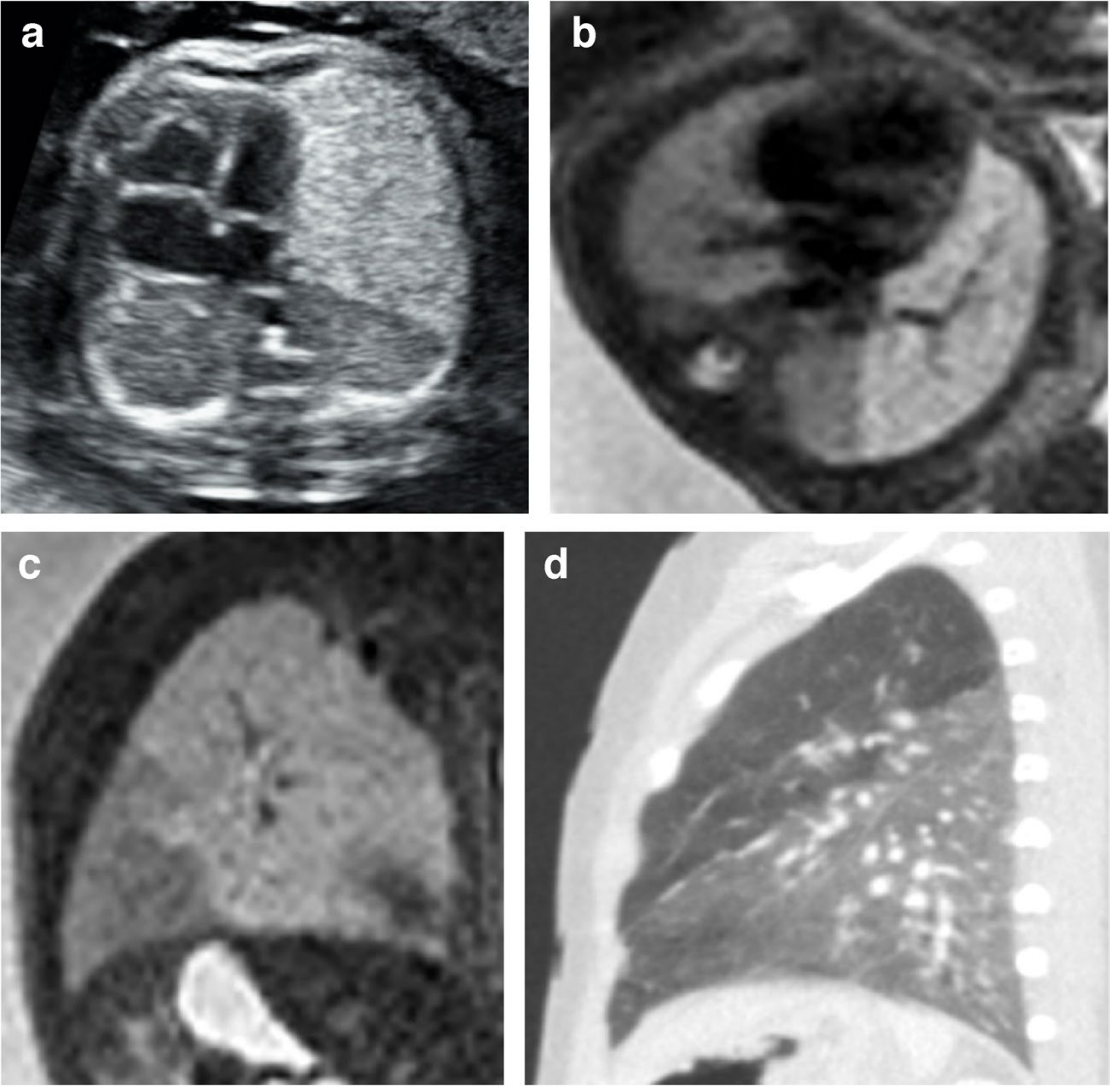
**a**–**c **Congenital lobar overinflation in a 29-week gestational age male fetus. Transverse ultrasound image (**a**) and axial (**b**) and sagittal (**c**) T2 single-shot magnetic resonance images (MRI) show asymmetry of the lungs; the left upper lobe is enlarged with hyperechogenicity on ultrasound and homogeneous high T2 signal on MRI. **d** A sagittal computed tomography image on lung window settings performed at 6 months of age demonstrates a hyperexpanded left upper lobe. Surgery two months later confirmed congenital lobar overinflation

The sonographic appearance of congenital lobar overinflation is usually a homogeneous hyperechoic solid mass that may present with hypervascularity [38]. Fetal MRI shows an expanded homogeneously solid lesion with high T2 signal without cysts (Fig. 9) [8]. Sonographic follow-up is indicated in order to exclude potential complications like hydrops, although this is rare [3].

## Conclusion

Congenital thoracic lesions are relatively uncommon pathologies that are usually first recognized in the fetal period. Prenatal US is the gold standard imaging technique for their diagnosis and follow-up. MRI can be used as an adjunct to better evaluate the anatomy and possible complications, to predict prognosis and to plan perinatal treatment. Physicians involved in the treatment of these abnormalities should be familiar with the prenatal imaging features and possible treatment options in order to provide appropriate parental counseling and to optimize perinatal outcome.

## Data Availability

The datasets generated during and/or analysed during the current study are available from the corresponding author on reasonable request.
